# *atp*D gene sequencing, multidrug resistance traits, virulence-determinants, and antimicrobial resistance genes of emerging XDR and MDR-*Proteus mirabilis*

**DOI:** 10.1038/s41598-021-88861-w

**Published:** 2021-05-04

**Authors:** Abdelazeem M. Algammal, Hany R. Hashem, Khyreyah J. Alfifi, Helal F. Hetta, Norhan S. Sheraba, Hazem Ramadan, Reham M. El-Tarabili

**Affiliations:** 1 Department of Bacteriology, Immunology, and Mycology, Faculty of Veterinary Medicine, Suez Canal University, Ismailia, 41522 Egypt; 2 Department of Microbiology and Immunology, Faculty of Pharmacy, Fayoum University, Fayoum, 63514 Egypt; 3 Department of Biology, Faculty of Science, Tabuk University, Tabuk, 7149 Saudi Arabia; 4 Department of Medical Microbiology and Immunology, Faculty of Medicine, Assuit University, Assuit, 71515 Egypt; 5 VACSERA, the Holding Company for Biological Products and Vaccines, Giza, 12511 Egypt; 6 Hygiene and Zoonoses Department, Faculty of Veterinary Medicine, Mansoura University, Mansoura, 35516 Egypt

**Keywords:** Antimicrobials, Bacteriology, Biofilms, Pathogens

## Abstract

*Proteus mirabilis* is a common opportunistic pathogen causing severe illness in humans and animals. To determine the prevalence, antibiogram, biofilm-formation, screening of virulence, and antimicrobial resistance genes in *P. mirabilis* isolates from ducks; 240 samples were obtained from apparently healthy and diseased ducks from private farms in Port-Said Province, Egypt. The collected samples were examined bacteriologically, and then the recovered isolates were tested for *atp*D gene sequencing, antimicrobial susceptibility, biofilm-formation, PCR detection of virulence, and antimicrobial resistance genes. The prevalence of *P. mirabilis* in the examined samples was 14.6% (35/240). The identification of the recovered isolates was confirmed by the *atp*D gene sequencing, where the tested isolates shared a common ancestor. Besides, 94.3% of *P. mirabilis* isolates were biofilm producers. The recovered isolates were resistant to penicillins, sulfonamides, β-Lactam-β-lactamase-inhibitor-combinations, tetracyclines, cephalosporins, macrolides, and quinolones. Using PCR, the retrieved strains harbored *atp*D*, ure*C*, rsb*A*,* and *zap*A virulence genes with a prevalence of 100%, 100%, 94.3%, and 91.4%, respectively. Moreover, 31.4% (11/35) of the recovered strains were XDR to 8 antimicrobial classes that harbored *bla*_TEM_*, bla*_OXA-1_*, bla*_CTX-M_*, tet*A*,* and *sul*1 genes. Besides, 22.8% (8/35) of the tested strains were MDR to 3 antimicrobial classes and possessed *bla*_TEM_*, tet*A*,* and *sul*1genes. Furthermore, 17.1% (6/35) of the tested strains were MDR to 7 antimicrobial classes and harbored *bla*_TEM_*, bla*_OXA-1_*, bla*_CTX-M_*, tet*A, and* sul*1 genes. Alarmingly, three strains were carbapenem-resistant that exhibited PDR to all the tested 10 antimicrobial classes and shared *bla*_TEM_, *bla*_OXA-1_, *bla*_CTX-M_, *tet*A, and *sul*1 genes. Of them, two strains harbored the *bla*_NDM-1_ gene, and one strain carried the *bla*_KPC_ gene*.* In brief, to the best of our knowledge, this is the first study demonstrating the emergence of XDR and MDR-*P.mirabilis* in ducks. Norfloxacin exhibited promising antibacterial activity against the recovered XDR and MDR-*P. mirabilis*. The emergence of PDR, XDR, and MDR-strains constitutes a threat alarm that indicates the complicated treatment of the infections caused by these superbugs.

## Introduction

The genus *Proteus* includes Gram-negative, moderate-sized, non-sporulated, and motile rods. *Proteus mirabilis* is one of the most prevalent *Proteus* species. *P. mirabilis* normally inhabits the intestinal tract of both humans and animals as normal flora. Besides, it's a ubiquitous environmental microorganism widely distributed in nature^1^. *P. mirabilis* is known as an opportunistic bacterial pathogen that incriminated in community-acquired infections, food-borne infections, serious nosocomial infections, and urinary tract infections in humans^2–5^. Furthermore, several recent studies proved the existence of *P. mirabilis* in animals and poultry. The molecular typing of *P. mirabilis* from human and animal origins revealed that the animal strains could be transmitted to humans^6–8^.

The emergence of multidrug-resistant bacterial pathogens is reflected as a public health risk. Several investigations reported the occurrence of MDR pathogens from different origins including humans, animals, birds, fish, and food^9–18^. The emergence of extended-spectrum β-lactam resistant *Proteus* species had been reported for the first time in 1987 that is considered a thoughtful public health concern globally^19^. Besides, the existence of MDR-*Proteus* species was reported in previous studies^6,20,21^.

The antimicrobial resistance genes could be transmitted to *P. mirabilis* from other resistant pathogens in the environment and the gastrointestinal tract, especially the extended-spectrum β-lactamase genes including; *bla*_TEM_ gene: encoded for penicillins-resistance, *bla*_CTX_ gene: encoded for cephalosporins-resistance, *bla*_NDM1_ gene: encoded for carbapenem-resistance, and *bla*_OXA-1_ gene: encoded for and piperacillin and cephalosporins-resistance^4,22^. *P. mirabilis* is known as a common biofilm producer. The bacterial biofilm protects the bacteria from the phagocytic cells, the environmental stresses, and different antimicrobial agents. Moreover, it is considered a frequent source of infection^3,5^.

PCR is a rapid and specific reliable epidemiological tool used for screening virulence and antimicrobial resistance genes in certain bacterial pathogens. The *atp*D gene is one of the most conserved genes in *Proteus* species that encodes for ATP synthase β-subunit. The pathogenicity of *P. mirabilis* is regulated by several virulence determinants that are controlled by multiple virulence genes such as *ure*C, *zap*A, and *rsb*A virulence genes. The *rsb*A gene is responsible for swarming modulation in *Proteus* species. Moreover, the *ure*C gene is the principal gene responsible for urease enzyme production that plays a major role in stone formation in kidneys or bladder during urinary tract infections. Furthermore, the *zap*A gene is encoded for protease production, especially IgA protease^2,5,6,23^.

This study is aimed to investigate the prevalence, *atp*D gene sequencing, antibiogram, PCR detection of virulence genes (*ure*C, *zap*A, and *rsb*A), and antimicrobial resistance genes (*bla*_TEM_, *bla*_CTX_, *bla*_NDM-1_, *bla*_KPC_, *bla*_OXA-1_, *sul*1, and *tet*A) of emerging *P. mirabilis* in ducks.

## Material and methods

### Animal ethics

The study was carried out in compliance with the ARRIVE guidelines. All methods were performed according to relevant guidelines and regulations. Handling of birds and all the experimental protocols conducted by well-trained scientists and were approved by the Animal Ethics Review Committee of Suez Canal University (AERC-SCU), Egypt.

### Sampling

Approximately, 240 specimens were obtained from apparently healthy (*n* = 40) and diseased ducks (*n* = 40) from private duck commercial farms (Muscovy duck with average age 70 days) at Port-Said Province, Egypt (From May 2020 to August 2020). Tracheal and cloacal swabs were collected from live birds, while the internal organs were collected separately under complete aseptic conditions from freshly dead and slaughtered ducks as illustrated in Table1. Diseased ducks exhibited diarrhea and respiratory manifestations. Specimens were collected in peptone water (Oxoid, UK) and rapidly transmitted to the lab as soon as possible for bacteriological examination.

**Table 1:** Various types of samples obtained from apparently healthy and diseased ducks.

Types of samples	Duck condition			
	Apparently healthy *n* = 40		Diseased ducks *n* = 40	
	Live *n* = 20	Freshly slaughtered *n* = 20	Live *n* = 20	Dead *n* = 20
Tracheal swabs *n* = 40	20	–	20	–
Cloacal swabs *n* = 40	20	–	20	–
Liver *n* = 40	–	20	–	20
Heart *n* = 40	–	20	–	20
Lung *n* = 40	–	20	–	20
Gizzard *n* = 40	–	20	–	20
Sub-total	40	80	40	80
Total	240			

### Isolation and identification of *P. mirabilis*

The obtained samples were enriched in peptone water (Oxoid, Hampshire, UK) at 37 °C for 24 h. A loopful from the enriched broth was streaked on XLD, 5% sheep blood agar, MacConkey agar, and TSI (Oxoid, Hampshire, UK), then left incubated at 37 °C for 24–48 h. The identification of suspected colonies was performed according to their culture characters, swarming activity, hemolytic activity, morphological characteristics using Gram's-staining, and biochemical characters as described by Quinn^24^. Moreover, the identification of *P. mirabilis* was confirmed by the PCR detection of the *atp*D gene as described by Bi^25^ (Table 2), followed by gene sequencing of the PCR products.

**Table 2:** The oligonucleotides sequences and thermal-cycling conditions of PCR assay.

Genes	Oligonucleotides sequences	Amplified product (bp)	PCR conditions (35 cycles)			References
			Denaturation	Annealing	Extension	
*atp*D	GTATCATGAACGTTCTGGGTAC TGAAGTGATACGCTCTTGCAG	595	94 °C 30 s	58 °C 40 s	72 °C 45 s	^25^
*ure*C	GTTATTCGTGATGGTATGGG ATAAAGGTGGTTACGCCAGA	317	94 °C 30 s	56 °C 40 s	72 °C 40 s	^30^
*rsb*A	TTGAAGGACGCGATCAGACC ACTCTGCTGTCCTGTGGGTA	467	94 °C 30 s	58 °C 40 s	72 °C 45 s	^30^
*zab*A	ACCGCAGGAAAACATATAGCCC GCGACTATCTTCCGCATAATCA	540	94 °C 30 s	59 °C 40 s	72 °C 45 s	^30^
*tet*A	GGTTCACTCGAACGACGTCA CTGTCCGACAAGTTGCATGA	576	94 °C 30 s	50 °C 40 s	72 °C 45 s	^31^
*sul*1	CGGCGTGGGCTACCTGAACG GCCGATCGCGTGAAGTTCCG	433	94 °C 30 s	54 °C 40 s	72 °C 45 s	^32^
*bla*_KPC_	ATGTCACTGTATCGCCGTCT TTACTGCCCGTTGACGCCC	892	94 °C 1 min	55 °C 1 min	72 °C 1 min	^33^
*bla*_NDM-1_	GGCGGAATGGCTCATCACGA CGCAACACAGCCTGACTTTC	287	94 °C 30 s	55 °C 30 s	72 °C 30 s	^33^
*bla*_CTX-M_	ATG TGC AGY ACC AGT AAR GTK ATG GC TGG GTR AAR TAR GTS ACC AGA AYC AGC GG	593	94 °C 30 s	54 °C 40 s	72 °C 45 s	^34^
*bla*_OXA-1_	ATATCTCTACTGTTGCATCTCC AAACCCTTCAAACCATCC	619	94 °C 30 s	54 °C 40 s	72 °C 45 s	^35^
*bla*_TEM_	ATCAGCAATAAACCAGC CCCCGAAGAACGTTTTC	516	94 °C 30 s	54 °C 40 s	72 °C 45 s	^35^

### The *atp*D gene sequencing and phylogenetic analyses

Since the retrieved isolates exhibited harmony in their phenotypic and biochemical characteristics: the PCR products of 5 randomly selected isolates were purified with a QIAquick PCR-Product extraction kit (QIAGEN Sciences Inc., Germantown, MD, USA) and sent for direct sequencing using the same set of primers. The sequencing was carried out using the Bigdye Terminator V3.1 cycle sequencing kit (Thermo Fisher Scientific, Waltham, MA, USA). The sequencing was performed using the Applied Biosystems 3130 genetic analyzer (HITACHI, Japan), and the retrieved sequences were deposited in the GenBank with accession numbers: MW357650, MW357651, MW357652, MW357653, and MW357654. To detect the sequence identity to GenBank accessions, the BLAST analysis (Basic Local Alignment Search Tool) was done. The phylogenetic tree was generated by the MegAlign module of LasergeneDNAStar version 12.1 using maximum likelihood, neighbor-joining, and maximum parsimony in MEGA6^26^.

### Antimicrobial susceptibility testing of *P. mirabilis*

The disc diffusion method was carried out to investigate the antibiogram of the obtained *P. mirabilis* isolates using Mueller-Hinton agar (Oxoid, Hampshire, UK). Fifteen antimicrobial agents were involved; colistin sulfate (CT) (10 μg), ceftazidime (CAZ) (30 μg), amoxicillin (AMX) (10 μg), norfloxacin (NOR) (10 μg), piperacillin (PRL) (10 μg), amoxicillin-clavulanic acid (AMC) (30 μg), imipenem (IPM) (10 μg), nalidixic acid (ND) (30 μg), ampicillin (AMP) (10 μg ), cefotaxime (30 μg) (CTX), erythromycin (E) (15 μg), ampicillin-sulbactam(SAM) (30 μg), meropenem (MEM) (10 μg), trimethoprim-sulfamethoxazole (SXT) (19:1 μg), and doxycycline (DOX) (10 μg) (Oxoid, UK). The test was performed using *E. coli*-ATCC 35218 as a control strain. The diameter of the inhibition zone was estimated as described by CLSI^27^. The phenotypic resistance patterns are categorized into PDR, XDR, and MDR according to Magiorakos^28^.

### Estimation of the biofilm formation in the isolated *P. mirabilis*

Estimation of biofilm formation was carried out in glass test tubes as previously described by Kadam^29^. Briefly, each *P. mirabilis* strain was inoculated in tryptic soy broth (Oxoid, Hampshire, UK), and left incubated overnight at 28 °C without shaking. Negative control experiments were carried out with sterile broth. After discarding the broth, the incubated tubes were stained with 1% crystal violet (to observe cells attached to the test tube) and were incubated for 15 min. Then, the tubes were washed with sterile distilled water. The test was repeated three times for each strain. Positive results indicated by the formation of purple biofilms.

### PCR detection of virulence and antimicrobial resistance genes in the retrieved *P. mirabilis*

The PCR-based detection of *ure*C, *zap*A, and *rsb*A virulence genes and *bla*_TEM,_ *bla*_CTX_, *bla*_NDM-1,_ *bla*_KPC_, *bla*_OXA-1_, *sul*1, and *tet*A antimicrobial resistance genes was performed. Extraction of bacterial DNA was carried out according to the descriptions of the QIAamp DNA Mini Kit (QIAGEN Sciences Inc., Germantown, MD, USA/ Cat. No. ID 51326). Accordingly, the reaction volume was 50 μl as follows: 5 μl of 10 × PCR reaction buffer, 1 μl 200 μM (of each dNTP) of dNTP mix (10 mM), 4 μl of bacterial-DNA, 0.4 μl 2 U of Taq DNA Polymerase (5 U/μl), 30 pmol of each used primer (0.1–0.6 μM), and then Sterile ddH_2_O was added up to 50 μl. Positive control strains (kindly supported by the Biotechnology Center of AHRI, Egypt) were involved in all PCR reactions. Besides, a reaction without a DNA-template was used as a negative control. Oligonucleotides-sequences (Thermo Fisher Scientific, Waltham, MA, USA) and the thermal-cycling protocols are described in Table 2. The agar gel electrophoresis was carried out for the separation of the obtained PCR-products using 1.5% agarose stained with ethidium bromide 0.5 μg/ml and followed by photographing the gel.

### Statistical analyses

The obtained findings were analysed using the Chi-square test (SAS software, version 9.4, SAS Institute, Cary, NC, USA) (Significance-level; *P* < 0.05). Besides, the correlation coefficient and the non-parametric Wilcox signed-rank test were performed using R-software (version 4.0.2; https//www.r-project.org/).

## Results

### Phenotypic characteristics and prevalence of *P. mirabilis* in the examined samples

**Figure 1:**
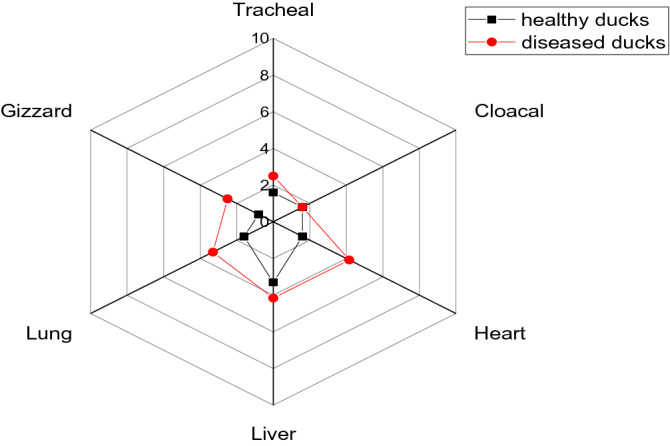
The radar illustrates the prevalence of *P. mirabilis* in different examined samples of apparently healthy and diseased ducks.

The recovered colonies are red with black center on XLD, pale colonies (non-lactose fermenter) on MacConkey agar, black colonies on TSI (H_2_S producer), hemolytic on blood agar, and undergo the characteristic swarming activity*.* Biochemically, the retrieved isolates were positive for catalase, H_2_S production, urease, methyl red, and citrate utilization tests, while are negative for oxidase, lactose fermentation, indole, and Voges-Proskauer tests. The prevalence of *P. mirabilis* among the examined birds was 25% (20/80); the prevalence was 15% (6/40) in the examined apparently healthy ducks, while it was 35% (14/40) in the examined diseased ducks (Table 3). Concerning the distribution of *P. mirabilis* in the examined samples, the total prevalence of *P. mirabilis* was 14.6% (35/240); the prevalence of *P. mirabilis* was 10.8% (13/120) in the examined samples of apparently healthy ducks, while the prevalence was 18.3% (22/120) in the examined samples of diseased ducks. The most predominant infected organ was the liver, followed by the heart and lung. Statistically, there is a significant difference in the prevalence of *P. mirabilis* between the examined apparently healthy and diseased ducks (*P* < 0.05), whereas there is no significant difference (*P* > 0.05) among different examined samples (Table 4 and Fig. 1).

**Table 3:** Prevalence of *P. mirabilis* among the examined birds.

Bird condition	No of examined birds	Positive for *P. mirabilis*	
		No	%
Apparently healthy	Alive (*n* = 20)	2	10
	Freshly dead (*n* = 20)	4	20
	Subtotal	6	15 (6/40)
Diseased	Alive (*n* = 20)	5	25
	Freshly dead (*n* = 20)	9	45
	Subtotal	14	35(14/40)
Total	80	20	25 (20/80)

**Table 4:** Prevalence of *P. mirabilis* in different examined samples of apparently healthy and diseased ducks.

Type of sample *n* = 40 (for each type of samples)	*Proteus mirabilis*				Total (total number of samples = 240)	
	Apparently healthy ducks (No. of samples = 120)		Diseased ducks (No. of samples = 120)			
	N	%	N	%	N	%
Tracheal swabs	2	1.6	3	2.5	5	2.08
Cloacal swabs	2	1.6	2	1.6	4	1.66
Heart	2	1.6	5	4.16	7	2.9
Liver	4	3.3	5	4.16	9	3.75
Lung	2	1.6	4	3.3	6	2.5
Gizzard	1	0.8	3	2.5	4	1.66
Total	13	10.8	22	18.3	35	14.6
Chi square *P*-value	2.2308 0.8164		2 0.8491		3.2286 0.6648	
Wilcoxon test *p*-value	0.04123

### Sequence analysis of the *atp*D gene

**Figure 2:**
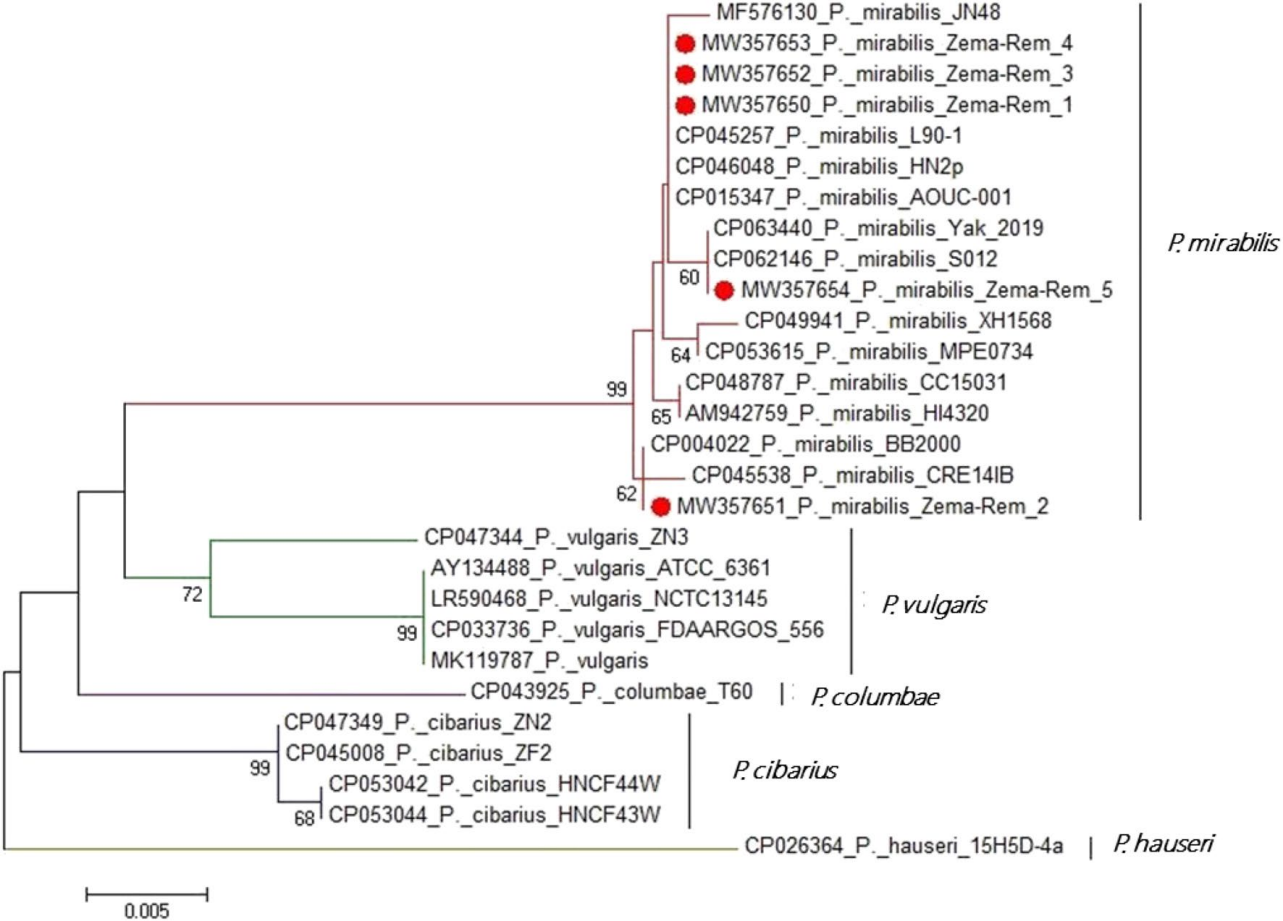
The phylogenetic analyses were based on the *atp*D gene sequencing. The phylogenetic tree illustrates the genetic relatedness of the retrieved *P. mirabilis* isolates and other relevant isolates deposited in the GenBank database. The tree topology was assessed by bootstrap analysis of 1000 replicates. The bacteria recovered in the present study are marked with solid red circles.

The *atp*D gene sequencing and the phylogenetic analysis proved that the tested *P. mirabilis* isolates (*n* = 5) shared a common ancestor. Moreover, the tested isolates showed high genetic identity to other strains of *P. mirabilis* such as *P. mirabilis* strain HI4320 of United Kingdom (Accession No. AM942759), *P. mirabilis* strain BB2000 of China (Accession No. MF576130), *P. mirabilis* strain BB2000 (Accession No. CP045538) and strain AOUC-001 (Accession No. CP015347) of Italy, and *P. mirabilis* strain BB2000 of USA (Accession No. CP004022) as illustrated in Fig. 2.

### Antibiogram and the phenotypic resistance patterns of the isolated *P. mirabilis*

**Figure 3:**
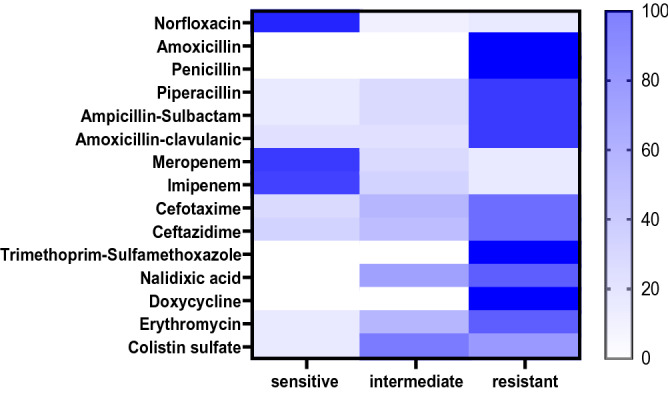
The heat-map illustrates the different degrees of susceptibility (sensitive, intermediate, and resistant) of the retrieved *P. mirabilis* to different tested antimicrobial agents.

The recovered *P. mirabilis* isolates exhibited remarkable resistance-patterns to various antimicrobial classes including; Penicillins: amoxicillin and penicillin (100%), and piperacillin (77.1%), β-Lactam-β-lactamase-inhibitor combination: ampicillin-sulbactam and amoxicillin-clavulanic acid (71.7%), Sulfonamides: trimethoprim-sulfamethoxazole (100%); Tetracyclines: doxycycline (100%), Quinolones: nalidixic acid (62.8%), Macrolides: erythromycin (62.8%), and Cephalosporins: ceftazidime and cefotaxime (57.1%). Moreover, the tested isolates displayed intermediate resistance to Polymyxins: colistin sulfate (51.4%). Besides, the retrieved isolates were sensitive to Fluoroquinolones: norfloxacin (85.7%), and Carbapenems: meropenem (77.1%), and imipenem (74.3%). Furthermore, 8.3% of the tested *P. mirabilis* isolates (*n* = 3) were found to be carbapenem-resistant strains (Table 5, Supplementary Table S1, and Fig. 3). Statistically, there is a significant difference (*P* < 0.05) in the susceptibility of the obtained *P. mirabilis* isolates to different tested antimicrobial agents. Moreover, the correlation-coefficient between various involved antimicrobial agents was estimated.

**Table 5:** Antibiogram of the isolated *P. mirabilis* strains (*n* = *35*).

Antimicrobial classes	Antimicrobial agents	*P. mirabilis* (*n* = 35)					
		S		I		R	
		N	%	N	%	N	%
Fluoroquinolones	Norfloxacin	30	85.7	2	5.7	3	8.6
Penicillins	Amoxicillin	0	0	0	0	35	100
	Penicillin	0	0	0	0	35	100
	Piperacillin	3	8.6	5	14.3	27	77.1
β -Lactam- β-lactamase-inhibitor combinations	Ampicillin-Sulbactam	3	8.6	5	14.3	27	77.1
	Amoxicillin-clavulanic acid	4	11.4	4	11.4	27	77.1
Carbapenems	Meropenem	27	77.1	5	14.3	3	8.6
	Imipenem	26	74.3	6	17.1	3	8.6
Cephalosporins	Cefotaxime	5	14.3	10	28.6	20	57.1
	Ceftazidime	6	17.1	9	25.7	20	57.1
Sulfonamides	Trimethoprim-Sulfamethoxazole	0	0	0	0	35	100
Quinolones	Nalidixic acid	0	0	13	37.1	22	62.8
Tetracyclines	Doxycycline	0	0	0	0	35	100
Macrolides	Erythromycin	3	8.6	10	28.6	22	62.8
Polymyxins	Colistin sulfate	3	8.6	18	51.4	14	40
Chi square		219.73		69.034		87.152	
*P* value		< 0.0001		< 0.0001		< 0.0001

**Figure 4:**
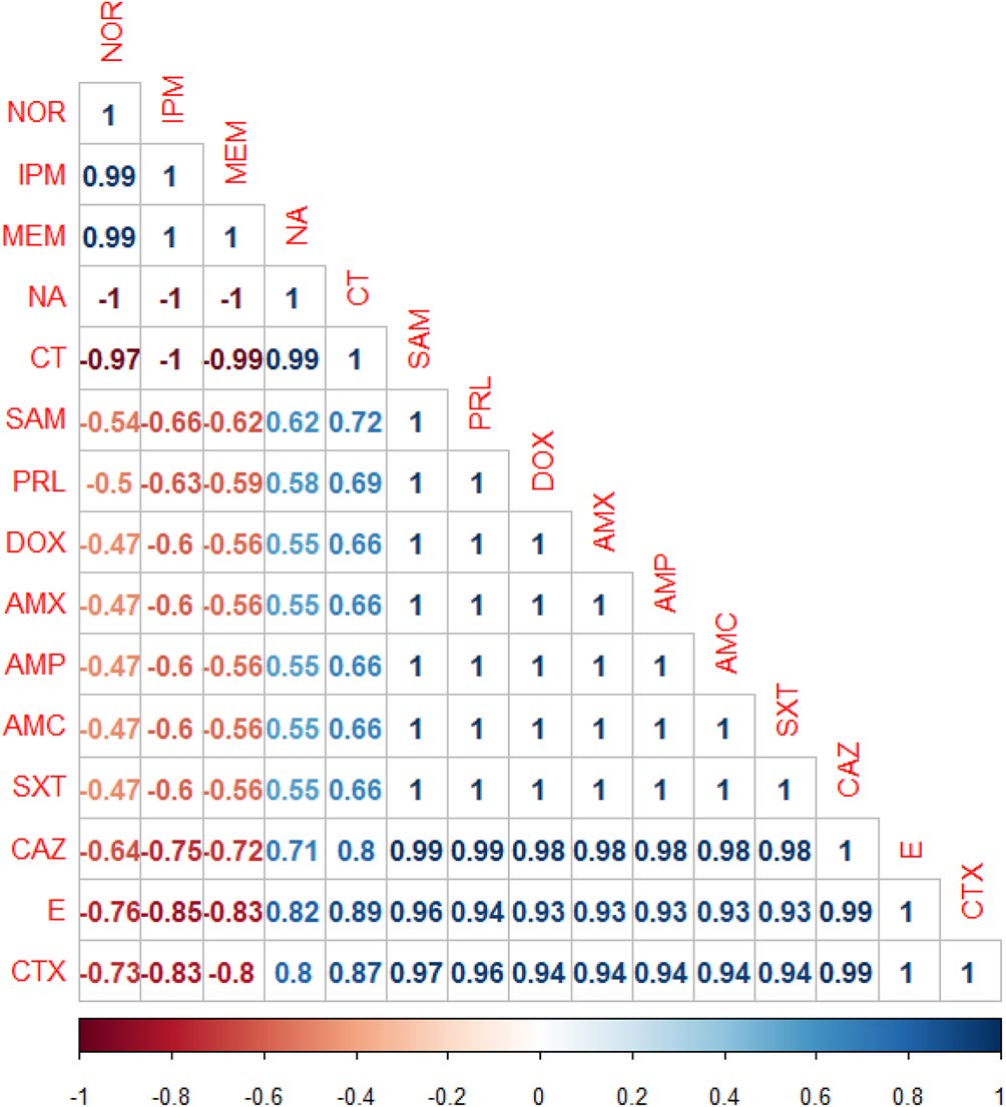
The heat-map illustrates the correlation-coefficient (r) among various antimicrobial agents. Blue and red color points to the positive and negative correlations, respectively.

Our findings proved a remarkable positive correlations (r = 0.5–1) between: NOR, IPM, and MEM (r = 0.99); E and CAZ (r = 0.99); CTX and CAZ (r = 0.99); SAM, PRL, and CAZ (r = 0.99); AMP, AMX, SXT, AMC, DOX, and CTX (r = 0.94); NA and CT (r = 0.99); AMP, AMX, SXT, AMC, DOX, and CAZ (r = 0.98); CTX and SAM (r = 0.98); SAM and CTX (r = 0.97); PRL and CTX (r = 0.96); E and SAM (r = 0.96); E and PRL (r = 0.94); AMP, AMX, SXT, AMC, DOX, and E (r = 0.93); E and CT (r = 0.89); CTX and CT (r = 0.87); as described in the heat-map (Fig. 4).

### The prevalence of biofilm formation among the recovered *P. mirabilis* strains

**Figure 5:**
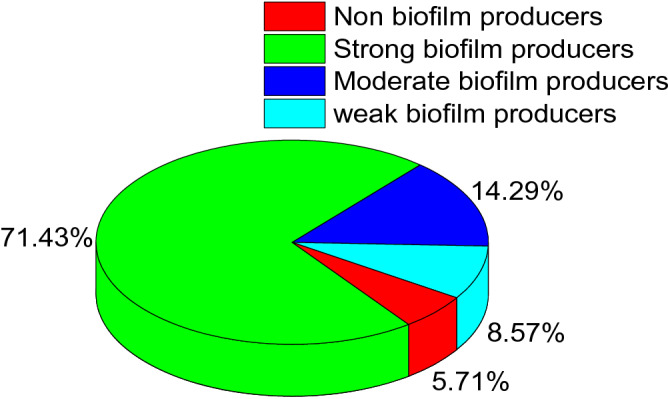
Illustrates the prevalence of biofilm-formation among the retrieved *P. mirabilis* strains. The percentage was calculated according to the total number of the retrieved isolates (*n* = 35).

Approximately 94.3% (33/35) of the isolated *P. mirabilis* strains were biofilm producers, while 5.7% (2/35) of the tested strains are non-biofilm producers. Of the biofilm producers (*n* = 33), 25 strains (75.8%) were strong biofilm producers, 5 strains (15.1%) were moderate biofilm producers, and 3 strains were weak biofilm producers (9.1%) as described in Fig. 5.

### Virulence and antimicrobial resistance genes of the recovered *P. mirabilis *strains

**Figure 6:**
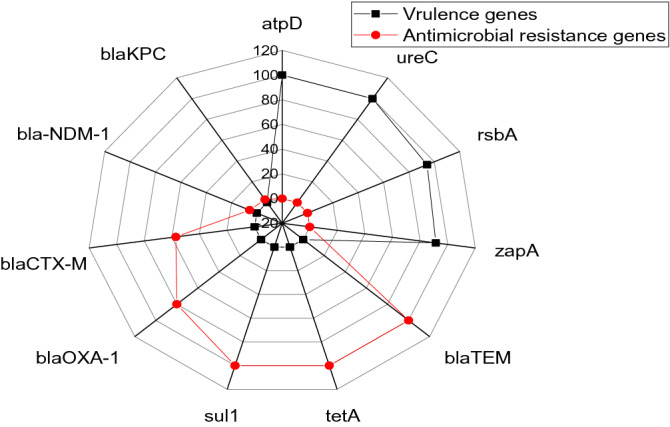
The radar reveals the frequency of virulence genes and antimicrobial resistant genes between the retrieved *P. mirabilis* strains.

The PCR revealed that the isolated *P. mirabilis* strains harbored *atp*D*, ure*C, *rsb*A*,* and *zap*A virulence genes with a prevalence of 100%, 100%, 94.3%, and 91.4%, respectively. Furthermore, the tested strains harbored *bla*_TEM_, *sul*1*, tet*A, *bla*_OXA-1_*, bla*_CTX-M_, *bla*_NDM-1_, and *bla*_KPC_ antimicrobial-resistance genes with a prevalence of 100%, 100%,100%, 80%, 57.1%, 5.7%, and 2.9%, respectively, as illustrated in Table 6 and Fig. 6. Statistically, there is no significant difference (*P* > 0.05) in the occurrence of virulence genes among the retrieved *P. mirabilis* strains, whereas there is a significant difference (*P* < 0.05) in the frequency of the antimicrobial resistance genes between the tested strains.

**Table 6:** Virulence and antimicrobial resistance genes that associated with the retrieved *P. mirabilis* (*n* = 35).

Types of genes		*N*	%	Chi square *P*-value
Virulence-determinant genes	*atp*D	35	100	0.2 0.9776
	*ure*C	35	100	
	*rsb*A	33	94.3	
	*zap*A	32	91.4	
Antimicrobial resistance genes	*bla*_TEM_	35	100	62.256 < 0.0001
	*tet*A	35	100	
	*sul*1	35	100	
	*bla*_OXA-1_	28	80	
	*bla*_CTX-M_	20	57.1	
	*bla*_-NDM-1_	2	5.7	
	*bla*_KPC_	1	2.9

### The correlation between the phenotypic and genotypic multidrug-resistance patterns in *P. mirabilis*

Our findings revealed that 31.4% (11/35) of the retrieved *P. mirabilis* strains are extensively drug-resistant (XDR: resistant to ≥ one agent in all but ≤ two antimicrobial classes) to 8 antimicrobial classes (Penicillins: amoxicillin, ampicillin, and piperacillin, β -Lactam- β-lactamase inhibitor combination: ampicillin-sulbactam, and amoxicillin-clavulanic acid, Cephalosporins: cefotaxime, and ceftazidime, Sulfonamides: trimethoprim-Sulfamethoxazole, Tetracyclines: doxycycline, Quinolones: nalidixic acid, Macrolides: erythromycin, and Polymyxins: colistin sulfate) and harbored *bla*_TEM_, *bla*_OXA-1_, *bla*_CTX-M_, *tet*A, and *sul*1 resistance genes. Furthermore, 22.8% (8/35) of the tested strains revealed multidrug resistance (MDR: resistant to ≥ one agent in ≥ 3 antimicrobial classes) to 3 antimicrobial classes (Tetracyclines: doxycycline, Penicillins: amoxicillin, and ampicillin, and Sulfonamides: trimethoprim-sulfamethoxazole) and possessed *tet*A, *bla*_TEM_, and *sul*1 resistance genes.

Besides, 17.1% (6/35) of the tested strains are MDR to 7 antimicrobial classes (Penicillins: amoxicillin, ampicillin, and piperacillin, β -Lactam- β-lactamase inhibitor combinations, Cephalosporins: cefotaxime, and ceftazidime, Sulfonamides: trimethoprim-Sulfamethoxazole, Tetracyclines: doxycycline, Quinolones: nalidixic acid, and Macrolides: erythromycin), and carried *bla*_TEM_, *bla*_OXA-1_, *bla*_CTX-M_, *tet*A, and *sul*1 resistance genes. Moreover, 14.2% (5/35) of the tested strains are MDR to 4 antimicrobial classes (Penicillins: amoxicillin, ampicillin, and piperacillin, β -Lactam- β-lactamase inhibitor combinations, Sulfonamides: trimethoprim-Sulfamethoxazole, and Tetracyclines: doxycycline) and harbored *bla*_TEM_, *bla*_OXA-1_, *tet*A, and *sul*1 resistance genes.

**Figure 7:**
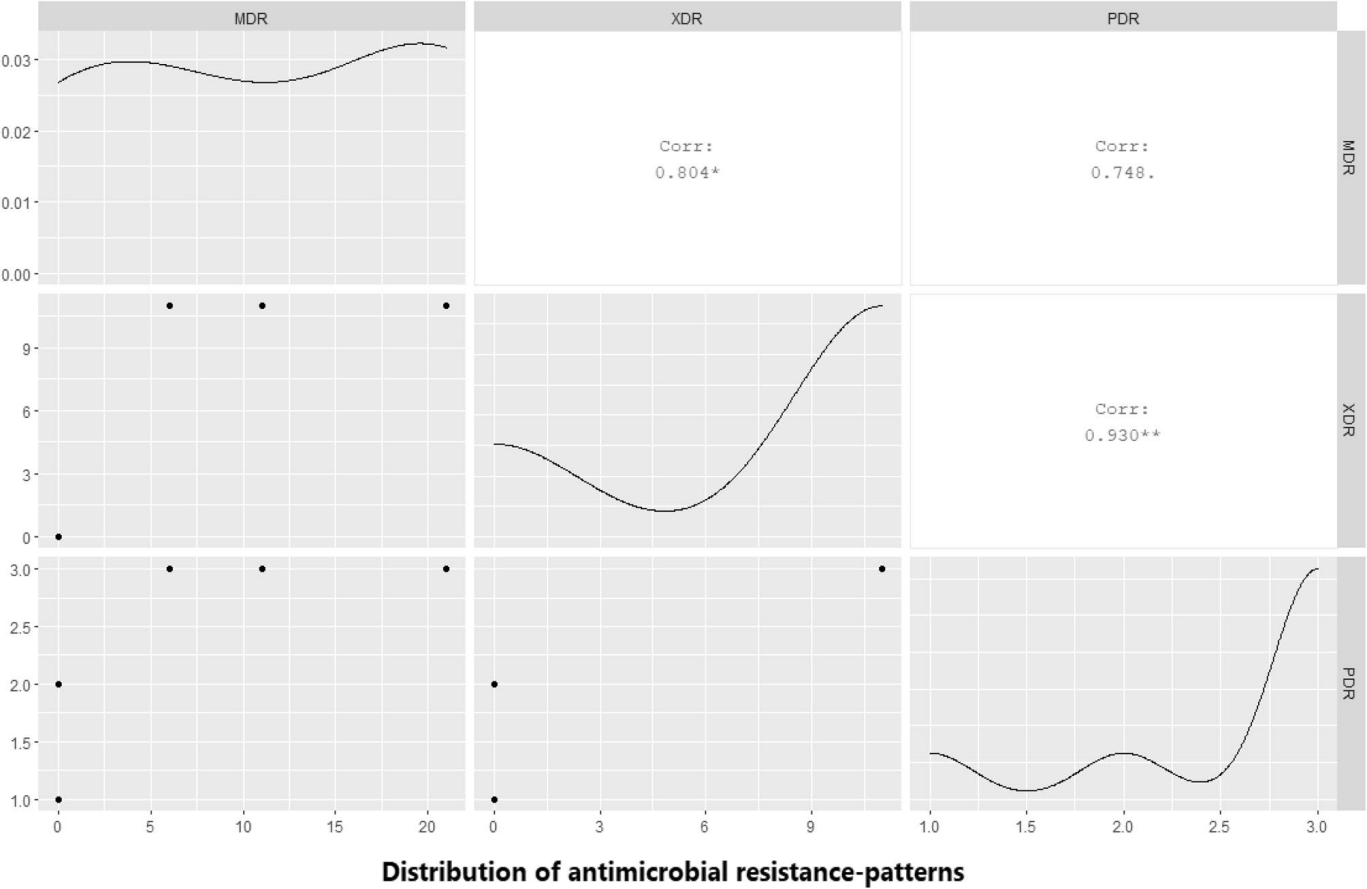
Illustrates the distribution of PDR, XDR, and MDR among the retrieved *P. mirabilis* strains.

Unfortunately, 3 strains are Pan-drug resistant (PDR) to all the tested 10 antimicrobial classes (Carbapenems: imipenem and meropenem, Fluoroquinolones: norfloxacin, Penicillins: amoxicillin, ampicillin, and piperacillin, β -Lactam- β-lactamase inhibitor combinations, Sulfonamides: trimethoprim-Sulfamethoxazole, Cephalosporins: cefotaxime, and ceftazidime, Tetracyclines: doxycycline, Quinolones: nalidixic acid, Macrolides: erythromycin, and Polymyxins: colistin sulfate); two strains harbored *bla*_TEM_*, bla*_OXA-1*,*_* bla*_CTX-M_*, bla*_NDM-1*,*_* tet*A and_*,*_* sul*1 genes, while one strain harbored *bla*_TEM_, *bla*_OXA-1_, *bla*_CTX-M_, *bla*_KPC_, *tet*A, and *sul*1 resistant genes as described in Table 7 and Fig. 7.

**Table 7:** The correlation between phenotypic and genotypic resistance patterns among the retrieved *P. mirabilis* (*n* = 35).

No. of strains	%	Type of resistance	In-vitro phenotypic resistance	The antimicrobial resistance genes
11	31.4	XDR	Penicillins: amoxicillin, ampicillin, and piperacillin	*bla*_TEM_, *bla*_OXA-1_, *bla*_CTX-M_, *tet*A, and *sul*1
			β-Lactam-β-lactamase inhibitor combination:	
			ampicillin-sulbactam, and amoxicillin-clavulanic acid	
			Cephalosporins: cefotaxime, and ceftazidime,	
			Sulfonamides: trimethoprim-Sulfamethoxazole	
			Tetracyclines: doxycycline	
			Quinolones: nalidixic acid	
			Macrolides: erythromycin	
			Polymyxins: colistin sulfate	
8	22.8	MDR	Penicillins: amoxicillin, and ampicillin	*bla*_TEM_, *tet*A, and *sul*1
			Tetracyclines: doxycycline	
			Sulfonamides: trimethoprim-sulfamethoxazole	
6	17.1	MDR	Penicillins: amoxicillin, ampicillin, and piperacillin	*bla*_TEM_, *bla*_OXA-1_, *bla*_CTX-M*,*_* tet*A, and *sul*1
			β -Lactam- β-lactamase inhibitor combinations	
			Cephalosporins: cefotaxime, and ceftazidime	
			Sulfonamides: trimethoprim-Sulfamethoxazole	
			Tetracyclines: doxycycline	
			Quinolones: nalidixic acid	
			Macrolides: erythromycin	
5	14.2	MDR	Penicillins: amoxicillin, ampicillin, and piperacillin	*bla*_TEM_, *bla*_OXA-1_, *tet*A, and *sul*1
			β -Lactam- β-lactamase inhibitor combinations	
			Sulfonamides: trimethoprim-Sulfamethoxazole	
			Tetracyclines: doxycycline	
2	5.7	PDR	Penicillins: amoxicillin, ampicillin, and piperacillin	*bla*_TEM_, *bla*_OXA-1_, *bla*_CTX-M_, *bla*_NDM-1_, *tet*A, and *sul*1
			β -Lactam- β-lactamase inhibitor combinations	
			Cephalosporins: cefotaxime, and ceftazidime	
			Fluroquinolones: Norfloxacin	
			Sulfonamides: trimethoprim-Sulfamethoxazole	
			Tetracyclines: doxycycline	
			Quinolones: nalidixic acid	
			Macrolides: erythromycin	
			Polymyxins: colistin sulfate	
			Carbapenems: imipenem and meropenem	
2	5.7	MDR	Penicillins: amoxicillin, ampicillin, and piperacillin	*bla*_TEM_, *bla*_OXA-1,_ *tet*A_,_ and *sul*1
			β -Lactam- β-lactamase inhibitor combinations	
			Sulfonamides: trimethoprim-Sulfamethoxazole	
			Tetracyclines: doxycycline	
			Quinolones: nalidixic acid	
			Macrolides: erythromycin	
1	2.9	PDR	Penicillins: amoxicillin, ampicillin, and piperacillin	*bla*_TEM_, *bla*_OXA-1_, *bla*_CTX-M_, *bla*_KPC_, *tet*A_,_ and *sul*1
			β -Lactam- β-lactamase inhibitor combinations	
			Cephalosporins: cefotaxime, and ceftazidime	
			Fluroquinolones: Norfloxacin	
			Sulfonamides: trimethoprim-Sulfamethoxazole	
			Tetracyclines: doxycycline	
			Quinolones: nalidixic acid	
			Macrolides: erythromycin	
			Polymyxins: colistin sulfate	
			Carbapenems: imipenem and meropenem

**Figure 8:**
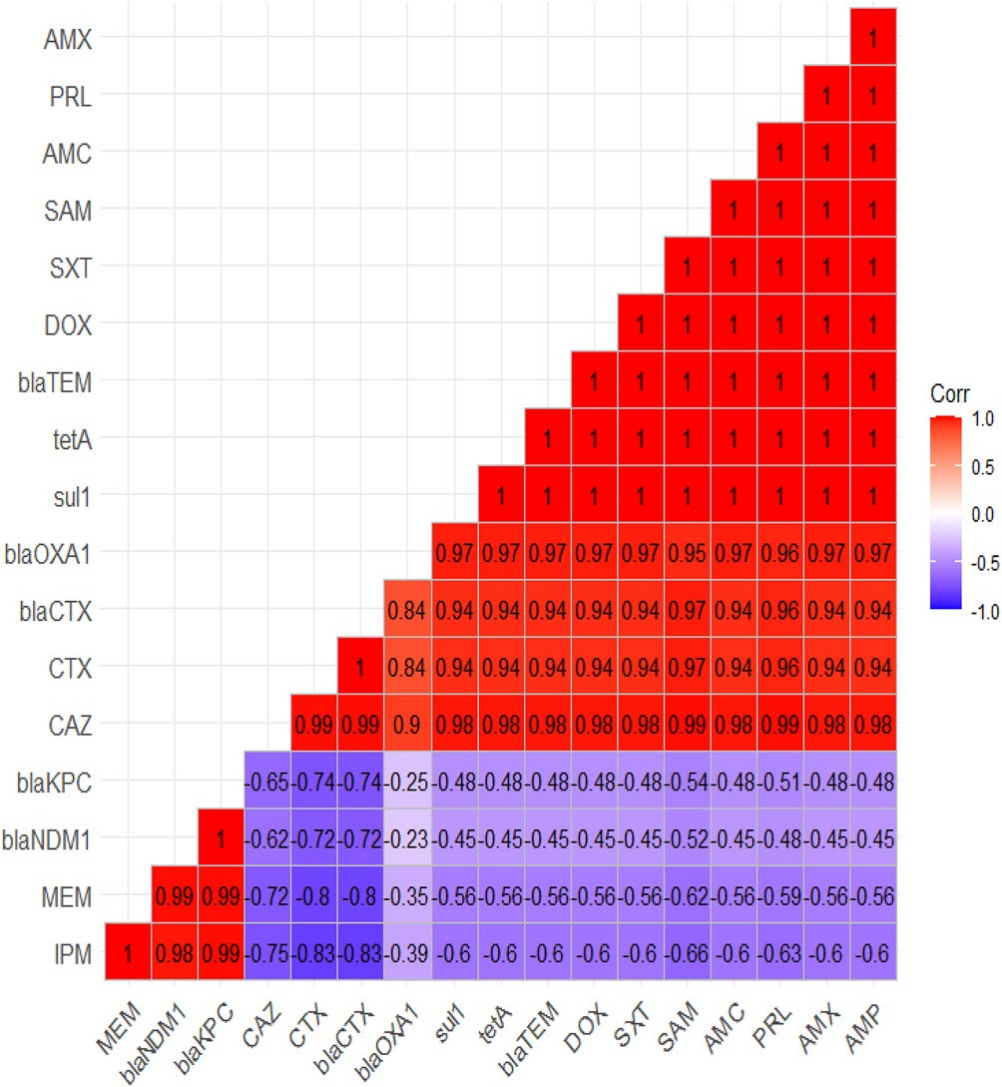
The Heat-map demonstrates the correlation-coefficient (r) between different tested antimicrobial agents and the antimicrobial resistance genes.

The correlation-coefficient (r) was estimated between different tested antimicrobial agents and the antimicrobial resistance genes. our findings proved a remarkable positive correlations between: (r = 0.5–1); *sul*1 gene and SXT (r = 1); *tet*A gene and DOX (r = 1), *bla*_CTX-M_ gene and CTX (r = 1); *bla*_CTX-M_ gene and CAZ (r = 0.99); *bla*_KPC_ gene, MEM, and IPM (r = 0.99); *bla*_NDM-1_ gene, MEM, and IPM (r = 0.99); *bla*_TEM_ gene_,_ AMX, and AMP (r = 0.99); *bla*_OXA-1_ gene, AMC, AMX, and AMP (r = 0.97); *bla*_OXA-1_ gene and PRL (r = 0.96); *bla*_OXA-1_ gene and SAM (r = 0.95); *bla*_OXA-1_ gene and CAZ (r = 0.90); *bla*_OXA-1_ gene and CTX (r = 0.84); *bla*_CTX-M_ gene and *bla*_OXA-1_ gene (r = 0.84); as illustrated in the heat-map (Fig. 8).

## Discussion

*Proteus mirabilis* is frequently incriminated in food-borne infections and urinary tract infections in humans. Few studies are concerning the emergence of *P. mirabilis* in birds. The current study was directed to investigate the prevalence, *atp*D gene sequencing, antimicrobial-resistance profiles, PCR-based detection of virulence genes (*ure*C, *zap*A, and *rsb*A), and antimicrobial resistance genes (*bla*_TEM_, *bla*_CTX_, *bla*_KPC_, *bla*_NDM-1_, *bla*_OXA-1_, *sul*1, and *tet*A) of emerging *P. mirabilis* in ducks.

The bacteriological examination evidenced that the prevalence of *P. mirabilis* in the examined samples was 14.6% (35/240). Besides, there is no ambivalence in the phenotypic characteristics of the retrieved *P. mirabilis* strains that revealed a significant harmony between the isolates: red colonies with black center on XLD, pale colonies (non-lactose fermenter) on MacConkey agar, black colonies on TSI, and undergo a characteristic swarming activity*.* Biochemically: the retrieved isolates are positive for catalase, H_2_S, urease, methyl-red, and citrate utilization tests, whereas they are negative for oxidase, lactose fermentation, indole, and Voges-Proskauer tests. These results are in agreement with those obtained by Lei^6^ and Reich^7^. In the present study, *P. mirabilis* was isolated from the internal organs of the examined birds in a pure form suggesting that the retrieved isolates were the primary bacterial cause of these infections in ducks. These results were supported by the previous findings that were reported by Barbour^36^ and Yeh^37^. *P. mirabilis* is a ubiquitous pathogen widely distributed in the environment^38^. *P. mirabilis* is an opportunistic pathogen that is incriminated in various infections in humans, animals, and poultry. Recently, several studies reported the emergence of *P. mirabilis* in food-producing animals, especially poultry^36,38,39^.

In the present study, *P. mirabilis* could be isolated from the internal organs of both apparently healthy and diseased birds. *P. mirabilis* is an opportunistic microorganism that normally inhabits the alimentary tract of birds, animals, and humans. The microorganism could escape from the intestinal tract and reach other internal organs. Thus, it could be responsible for other illnesses associated with the spread of *P. mirabilis* to other internal organs, and in severe cases, it could cause sepsis. In the meantime, the development of the clinical signs depends mainly on the onset of the disease as well as the immune status of the bird^40^.

The *atp*D gene phylogenetic analysis revealed that the tested *P. mirabilis* isolates (*n* = 5) are shared a common ancestor. Besides, they exhibited high genetic identity with other *P. mirabilis* strains of human origin that were previously isolated in Italy^41,42^, China^43^, USA^44^, and United Kingdom^45^. Our findings conceived the epidemiological map and emphasized the zoonotic impact of *P. mirabilis* that is considered a public health threat.

Concerning the in-vitro antimicrobial-resistance profiles; the recovered *P. mirabilis* strains showed remarkable resistance-patterns to penicillins, β-Lactam β-lactamase-inhibitor combinations, cephalosporins, sulfonamides, tetracyclines, macrolides, and quinolones. The development of such resistant strains reflected as a public health alarm. Moreover, the retrieved strains were sensitive to norfloxacin (85.7%), meropenem (77.1%), and imipenem (74.3%). Our findings are consistent with those reported by Wong^46^ and Nahar^47^. The improper application of antimicrobial agents in the poultry industry and the ability of *P. mirabilis* to acquire the antimicrobial-resistant genes from other resistant pathogens are the major causes of the emergence of these MDR-strains. Unfortunately, *P. mirabilis* could resist various antimicrobial classes due to the presence of chromosomal antibiotic-resistant genes as well as the resistant-plasmids^37^.

The biofilm assay revealed that 94.3% (33/35) of the isolated *P. mirabilis* strains are biofilm producers. Our findings are consistent with those reported by Kwiecinska-Piróg^48^. The biofilm is one of the most important virulence determinants of bacteria. It preserves bacteria during adverse environmental conditions. Moreover, biofilm protects bacteria from phagocytosis, antibodies, and antibiotics. Besides, it plays a vital role in antimicrobial resistance. *P. mirabilis* produces biofilm in various environments includes: biological and non-biotic surfaces such as glass, silicone, and polystyrene. The formation of biofilm on the non-biotic surface is considered the main source of nosocomial infections^49,50^.

The PCR proved that the recovered *P. mirabilis* strains are virulent and harbored *atp*D*, ure*C*, rsb*A*,* and *zap*A virulence genes with a prevalence of 100%, 100%, 94.3%, and 91.4%, respectively. Our findings are nearly agreed with those reported by Pathirana^30^ and Sun^51^. The *atp*D gene is encoded for ATP synthase β-subunit for the production of ATP from ADP. The *atp*D gene is more conservative in *Proteus* species when compared with 16SrRNA^25^. Infections caused by *P. mirabilis* are controlled by several virulence-determinants that are regulated by specific virulence genes. IgA‐degrading proteases are commonly accompanied by the pathogenic strains of *P. mirabilis*. ZapA-protease could degrade IgG, IgA1, and IgA2. It is regulated by the *zap*A gene. *P. mirabilis* is frequently incriminated in urinary tract infections that are mediated by stone-formation due to the release of urease enzyme. Urease is a metalloenzyme that acts by increasing the pH of urine that induces crystal formation. The urease production is controlled by the *ure*C gene. Besides, the characteristic swarming activity of *P. mirabilis* is encoded by the *rsb*A gene. The *rsb*A gene expresses a membrane sensor that induces the production of extracellular polysaccharides. Also, it regulates the swarming phenomena and enhances the biofilm formation by *P. mirabilis*^23,50,52,53^.

Concerning the correlation between the phenotypic and genotypic antimicrobial resistance patterns; our findings revealed that 31.4% (11/35) of the retrieved *P. mirabilis* strains are XDR to 8 antimicrobial classes, and harbored *bla*_TEM_, *bla*_OXA-1_, *bla*_CTX-M_, *tet*A, and *sul*1 genes. Moreover, 22.8% (8/35) of the tested strains are MDR to 3 antimicrobial classes and possessed *bla*_TEM_, *tet*A, and *sul*1genes. Besides, 17.1% (6/35) of the tested strains are MDR to 7 antimicrobial classes and harbored *bla*_TEM_, *bla*_OXA-1_, *bla*_CTX-M_, *tet*A, and *sul*1 genes. The Extended-spectrum β-lactamases (ESBLs) were reported for the first time in 1983^54^. ESBLs are responsible for the hydrolysis of Broad-spectrum β-lactam antibiotics including penicillins, piperacillin, and cephalosporins. EBSLs are frequently produced by *Enterobacteriales*. Recently, *P. mirabilis* strains reported harboring various acquired antimicrobial resistance genes. The high prevalence of the *bla*_TEM_ gene among the recovered *P. mirabilis* strains enabling them to resist penicillins (amoxicillin and ampicillin). Moreover, the resistance to cephalosporins (cefotaxime, and ceftazidime) is mediated by the presence of the *bla*_CTX-M_ gene. The resistance to piperacillin is mainly attributed to the *bla*_OXA-1_ gene which also promoting the resistance to cephalosporins. Besides, both *bla*_OXA-1_ and *bla*_CTX-M_ genes synergistically enable *P. mirabilis* to resist the β-Lactam-β-lactamase-inhibitor-combinations^55,56^. In addition, *P. mirabilis* is frequently resistant to tetracyclines and sulfonamides due to the presence of *tet*A and *sul*1genes, respectively. On the other hand, *P. mirabilis* is usually susceptible to fluoroquinolones such as norfloxacin^57^. The polymyxins exert their effect by increasing the permeability of the Gram-negative bacterial cell membrane through displacing Mg2 + and Ca2 + from the lipid A content of LPS that results in leakage of the cell contents. The resistance to polymyxins is common in the mutant *P. mirabilis* due to the alteration of LPS that is controlled by the expression of the *ept*C gene and the modification of the L- Ara4N. However, several previous studies reported the sensitivity of some *P. mirabilis* isolates to polymyxins, especially those of animal origin as reported by Sun^51^. The *ept*C gene may be present but not expressed. Besides, the alterations of the LPS in the cell envelop occurs only in the mutant strains and varies among different strains of *P. mirabilis* as previously reported by McCoy^58^.

In the present study, three strains are carbapenem-resistant as well as PDR to all the tested ten antimicrobial classes and are sharing *bla*_TEM_, bla_OXA-1_, *bla*_CTX-M_, *tet*A, and *sul*1 genes. Of them, two strains harbored the *bla*_NDM-1_ gene, and one strain carried the *bla*_KPC_ gene*.* Globally, the emergence of carbapenem-resistance in *P. mirabilis* is relatively low; however, it inclines to increase over time. The carbapenem-resistance is attributed to the presence of *bla*_NDM-1_ and *bla*_KPC_ genes. The existence of the *bla*_KPC_ gene in *P. mirabilis* was recorded for the first time in a diabetic Patient in the USA in 2008^59^, followed by China in 2010^60^, and Brazil in 2015^61^. Moreover, the *bla*_NDM-1_ is recognized for the first time in *P. mirabilis* strain retrieved from urinary infection in France in 2012^62^ and followed by China in 2015^63^.

Concerning the correlation between the antimicrobial resistance genes and the virulence determinants, a previous study that was reported by Filipiak^64^ revealed an inversed correlation between the virulence factors and the presence of the resistance genes in the retrieved *P. mirabilis* strains. However, in the present study, the majority of the screened virulence genes were found in the recovered isolates. Besides, there is no significant difference in the distribution of the virulence genes among the retrieved isolates either the susceptible or the antimicrobial-resistant strains. These findings suggest that the *P. mirabilis* pathogenicity is not affected by the presence of antimicrobial resistance genes.

### Study limitations

Multilocus sequence typing (MLST) should be carried out to illustrate the genetic relatedness among the recovered *P. mirabilis* strains.

In conclusion, to the best of our knowledge, this is the first report regarding the emergence of XDR and MDR-*P. mirabilis* in ducks. *P. mirabilis* is more prevalent in diseased birds than the apparently healthy ones, and the liver is the most prominent infected organ. *P. mirabilis* is a common biofilm-producing pathogen. The recovered *P. mirabilis* isolates commonly harbor the *atp*D*, ure*C*, zap*A*,* and *rsb*A virulence genes. The retrieved *P. mirabilis* strains are extensively drug-resistant (XDR) or multidrug-resistant (MDR) to several antimicrobial classes (penicillins, β-Lactam-β-lactamase-inhibitor-combinations, cephalosporins, sulfonamides, tetracyclines, quinolones, macrolides, and polymyxins), and commonly harbored *bla*_TEM_*, bla*_OXA-1*,*_* bla*_CTX-M*,*_* tet*A_*,*_ and *sul*1 antimicrobial resistance genes. In-vitro, norfloxacin exhibited promising antibacterial activity against the recovered XDR and MDR-*P. mirabilis*. Furthermore, the emergence of carbapenem-resistant (harbored either *bla*_KPC_ or *bla*_NDM-1_ genes) and PDR-strains constitutes a threat alarm that indicates a complicated treatment of the diseases caused by such superbugs. Accordingly, it endorses the incessant surveillance of antimicrobial susceptibility testing as well as the limited and appropriate use of antibiotics in health and veterinary practices.

## Supplementary Information

Supplementary Information.
