# Supplementary material for: atpD gene sequencing, multidrug resistance traits, virulence-determinants, and antimicrobial resistance genes of emerging XDR and MDR-Proteus mirabilis
Source: Sci Rep. 2021 May 4;11:9476. doi: 10.1038/s41598-021-88861-w (PMC8096940; doi:10.1038/s41598-021-88861-w)
Supplement: Supplementary file 1 — Supplementary Information. [file 41598_2021_88861_MOESM1_ESM.docx]

**Supplementary Table 1.** The antimicrobial susceptibility of the recovered *P. mirabilis* isolates (*n*=35) to various tested antimicrobial agents

| ***P. mirabilis* isolates** | **Tested Antimicrobial Agents** | | | | | | | | | | | | | | |
| --- | --- | --- | --- | --- | --- | --- | --- | --- | --- | --- | --- | --- | --- | --- | --- |
|  | **PRL** | **AMX** | **AMP** | **AMC** | **SAM** | **SXT** | **IPM** | **MEM** | **CTX** | **CAZ** | **E** | **CT** | **NA** | **DOX** | **NOR** |
| **1** | R | R | R | R | R | R | I | I | R | R | R | R | R | R | S |
| **2** | R | R | R | R | R | R | S | S | R | R | R | I | R | R | S |
| **3** | R | R | R | R | R | R | I | I | R | R | R | R | R | R | S |
| **4** | R | R | R | R | R | R | S | S | R | R | R | I | R | R | S |
| **5** | R | R | R | R | R | R | R | R | R | R | R | R | R | R | R |
| **6** | S | R | R | S | S | R | I | I | S | S | S | S | I | R | S |
| **7** | R | R | R | R | R | R | R | R | R | R | R | R | R | R | R |
| **8** | R | R | R | R | R | R | S | S | R | R | R | R | R | R | I |
| **9** | I | R | R | S | S | R | I | I | S | S | S | S | I | R | S |
| **10** | R | R | R | R | R | R | R | R | R | R | R | R | R | R | R |
| **11** | I | R | R | S | S | R | S | S | S | S | S | S | I | R | S |
| **12** | R | R | R | R | R | R | I | I | R | R | R | R | R | R | I |
| **13** | I | R | R | S | I | R | S | S | S | S | I | I | I | R | S |
| **14** | R | R | R | R | R | R | S | S | R | R | R | I | R | R | S |
| **15** | R | R | R | R | R | R | I | S | S | S | I | I | I | R | S |
| **16** | R | R | R | R | R | R | S | S | R | R | R | R | R | R | S |
| **17** | R | R | R | R | R | R | S | S | I | S | I | I | I | R | S |
| **18** | R | R | R | R | R | R | S | S | R | R | R | R | R | R | S |
| **19** | I | R | R | I | I | R | S | S | I | I | I | I | I | R | S |
| **20** | R | R | R | R | R | R | S | S | R | R | R | I | R | R | S |
| **21** | R | R | R | R | R | R | S | S | I | I | I | I | I | R | S |
| **22** | I | R | R | I | I | R | S | S | I | I | I | I | I | R | S |
| **23** | R | R | R | R | R | R | S | S | R | R | R | R | R | R | S |
| **24** | R | R | R | R | R | R | S | S | I | I | R | I | R | R | S |
| **25** | R | R | R | R | R | R | S | S | I | I | I | I | I | R | S |
| **26** | R | R | R | R | R | R | S | S | R | R | R | R | R | R | S |
| **27** | S | R | R | I | I | R | S | S | I | I | I | I | I | R | S |
| **28** | S | R | R | I | I | R | S | S | I | I | I | I | I | R | S |
| **29** | R | R | R | R | R | R | S | S | R | R | R | R | R | R | S |
| **30** | R | R | R | R | R | R | S | S | R | R | R | I | R | R | S |
| **31** | R | R | R | R | R | R | S | S | I | I | R | I | R | R | S |
| **32** | R | R | R | R | R | R | S | S | R | R | R | R | R | R | S |
| **33** | R | R | R | R | R | R | S | S | I | I | I | I | I | R | S |
| **34** | R | R | R | R | R | R | S | S | R | R | R | R | R | R | S |
| **35** | R | R | R | R | R | R | S | S | R | R | R | I | R | R | S |

**S= Sensitive, I= Intermediate, and R= Resistant**
